# Waterproof Fabric with Copper Ion-Loaded Multicompartmental Nanoparticle Coatings for Jellyfish Repellency

**DOI:** 10.3390/pharmaceutics18010047

**Published:** 2025-12-30

**Authors:** Bo Wang, Muzi Yang, Ruiqian Yao, Haixia Zhao, Dengguang Yu, Lin Du, Shuaijun Zou, Yuanjie Zhu

**Affiliations:** 1 Department of Dermatology, Naval Medical Center, Naval Medical University, Shanghai 200052, China; wangbo_1993@smmu.edu.cn (B.W.); yaorq999@smmu.edu.cn (R.Y.); zhaohx@smmu.edu.cn (H.Z.); lynnie_du@smmu.edu.cn (L.D.); 2 Faculty of Naval Medicine, Naval Medical University, Shanghai 200433, China; yangmuzi@simm.ac.cn; 3 School of Materials & Chemistry, University of Shanghai for Science and Technology, Shanghai 200093, China

**Keywords:** copper ions, multicompartmental nanoparticles, jellyfish repellency, drug release, functional re-finishing of fabrics

## Abstract

**Background:** Effective prevention of jellyfish stings is crucial for human safety during marine activities. Traditional protective methods are often limited in terms of coverage area and duration of protection; **Methods**: This study designed and tested a novel jellyfish-repellent textile by coating waterproof polyester fabric with copper ion-loaded multicompartmental nanoparticles, which repel jellyfish by disrupting their cellular membranes and physiological functions. The nanoparticles were synthesized to enable spatial separation of components, enhance stability, and allow controlled copper ion release. They were applied to the fabric in one step via high-voltage electrostatic spray technology, followed by characterization using SEM and FT-IR. The copper sulfate release profile and nanoparticle adhesion were analyzed. Jellyfish-repellent efficacy was evaluated, along with biocompatibility tests including skin sensitization (Magnusson and Kligman method), skin irritation (Draize test), and cytotoxicity (MTT assay on L929 cells and human dermal fibroblasts). **Results**: SEM confirmed the formation of uniform multicompartmental nanoparticles with sizes ranging from 2.28 to 3.15 μm. FT-IR verified successful anchoring of Cu^2+^ ions to fabric fibers through coordination with hydroxyl groups. Drug release tests demonstrated water-triggered controlled release of copper ions lasting over 168 h, with nanoparticle retention rates exceeding 70% on all fabrics. The textile showed significant effectiveness in repelling jellyfish. Moreover, no apparent sensitization, irritation, or cytotoxicity was observed. **Conclusions**: A novel jellyfish-repellent textile was successfully developed using copper ion-loaded multicompartmental nanoparticles. This textile provides a promising solution for preventing jellyfish stings and contributes to the advancement of protective gear for marine activities.

## 1. Introduction

Jellyfish stings significantly threaten human marine activities such as swimming, diving, and fishing due to their venomous tentacles, which can cause severe pain, skin irritation, and potentially life-threatening allergic reactions [1]. The increasing frequency of jellyfish blooms, driven by climate change and overfishing, has heightened the need for effective sting prevention measures. Traditional methods, such as net barriers and topical creams, are limited in coverage and duration [1,2]. These materials provide physical barriers but lack active repellency [3,4].

Recent research indicates that chemicals such as copper sulfate can repel jellyfish; Ref. [5] leveraged the biocidal properties of copper ions, which disrupt cellular membranes and inhibit enzymatic activities in aquatic organisms. Copper ions have been effectively used as algicides and molluscicides in aquatic systems [6,7]. Previous studies indicate that copper ions can harm aquatic species such as jellyfish by disrupting their physiological functions [8,9,10,11]. However, their use as a repellent in nanoparticle systems remains unexplored, necessitating further research. Additionally, incorporating active chemicals into fabrics is difficult due to poor adhesion and rapid degradation [12,13]. Innovative solutions are needed to address these challenges and ensure reliable, long-lasting protection for those involved in marine activities.

Nanotechnology offers a versatile platform for biomedical applications due to its controllable size, shape, and composition, enabling precise functional modulation [14,15,16,17]. Drug release can be triggered by pH, temperature, or ionic strength changes, common in marine environments [18,19,20,21,22]. Nevertheless, research on using nanoparticles to enhance jellyfish repellency is limited, with most studies focusing on single-compartment nanoparticles with one active ingredient, potentially restricting long-term effectiveness and versatility [23,24]. Recently, multicompartmental nanoparticles have shown benefits over traditional single-compartment ones by allowing the spatial separation of components, which reduces interactions and enhances stability [25,26,27]. This study was designed to explore copper ion-loaded multicompartmental nanoparticle coatings (using copper acetate, copper sulfate, and copper chloride) as a new method for preventing jellyfish stings and to test their repellency effectiveness through laboratory and field experiments.

## 2. Materials and Methods

### 2.1. Agents and Materials

Polycaprolactone (PCL, Mn ~ 60,000) was purchased from Shanghai Aladdin Biochemical Technology Co., Ltd. (Shanghai, China). Thermoplastic polyurethane (TPU, 1185A, Mn ~ 100,000) was purchased from BASF SE, Germany. Copper salts (acetate, sulfate, and chloride) were all purchased from Shanghai Macklin Biochemical Technology Co., Ltd. (Shanghai, China). 2,2,2-trifluoroethanol (TFE, 99.5%) and N,N-dimethylformamide (DMF, AR, 99.5%) were both purchased from Shanghai Macklin Biochemical Technology Co., Ltd. Ethanol (ET, AR, 99.5%), dichloromethane (DCM, AR, 99.5%), and tetrahydrofuran (THF, AR, 99.5%) were all purchased from Sinopharm Chemical Reagent Co., Ltd. (Shanghai, China). Anhydrous calcium chloride (CaCl_2_, 99%) and magnesium chloride hexahydrate (MgCl_2_·6H_2_O, 99%), used for preparing artificial seawater, were purchased from Yuansheng Biotechnology (Shanghai) Co., Ltd. (Shanghai, China). Sodium chloride (NaCl, reagent-grade, 99.5%) and potassium chloride (KCl, 99.5%) were purchased from Shanghai Macklin Biochemical Technology Co., Ltd. All materials used in the experiments for this project were used as received without further purification.

### 2.2. Multicompartmental Nanoparticle Synthesis and Spraying

We chose 0.2 g of PCL for its biodegradability and compatibility and added 10 mL of 2,2,2-trifluoroethanol (TFE), along with 0.2 g of copper acetate, copper sulfate, or copper chloride (Working Solution 1). As a stabilizer and dispersant, 0.2 g of PVP was added to 10 mL of a mixed solution of ethanol (ET) and dichloromethane (DCM) (at a volume ratio of 1:1), with 0.2 g of CuCl_2_ added (Working Solution 2). A mass of 0.3 g of TPU was added to 10 mL of a mixed solution of tetrahydrofuran (THF) and N,N-dimethylformamide (DMF) (at a volume ratio of 1:1) (Working Solution 3) for its strong adhesion and film-forming properties. All solutions were stirred at 60 °C for 12 h, ultrasonically dispersed for 30 min, and individually loaded into 10 mL syringes, followed by spraying with the high-voltage electrostatic generator (voltage at 12 ± 1 kV; flow rates at 1 mL/h for the contact antibacterial layer (CA solution), 1 mL/h for the sustained-release drug-loaded layer (repellent/CA solution), and 2 mL/h for the adhesive layer (TPU solution; collection distance at 10 ± 1 cm; temperature controlled at 23 ± 3 °C; humidity maintained at 50 ± 10%).

### 2.3. Scanning Electron Microscopy (SEM) and Fourier Transform Infrared Spectroscopy (FTIR) of Multicompartmental Nanoparticles

The morphological analysis of the copper ion-loaded multicompartmental nanoparticles was carried out using SEM to visualize the surface morphology and internal structure. To investigate the interactions between the raw materials after they formed electrosprayed particles, the tested fabric samples were analyzed using an FTIR spectrometer (SHIMADZU, Kyoto, Japan). Each test consisted of 12 scans, with data collected in the range of 400 to 4000 cm^−1^ at a scan resolution of 4 cm^−1^.

### 2.4. Drug Release Profile Testing

An evaluation protocol for the drug release efficacy of repellent-loaded nanoparticles was established with reference to preliminary kinetic studies and referenced from standard drug release protocols. In detail, untreated fabric was cut into 5 cm × 5 cm squares and weighed, with the initial mass recorded as *W*_0_. After the electrospray finishing process, the fabric was weighed again, and the mass was recorded as *W_b_*. Three fabric samples were immersed in a beaker containing 1000 mL of simulated seawater and placed in a constant-temperature shaker incubator set at 25 °C with a reciprocating shaking speed of 60 rpm to simulate a marine environment. We collected 5 mL samples at time points of 1 min, 10 min, 1 h, 6 h, 12 h, 24 h, 36 h, 48 h, 60 h, 72 h, 96 h, 120 h, 144 h, and 168 h. After each sampling, an equal volume of fresh simulated seawater was immediately replenished.

The collected samples were analyzed using inductively coupled plasma optical emission spectrometry (ICP-OES) to determine the concentration of copper, and the percentage of drug release at each time point was calculated as follows:

$$P_{n}=\frac{V_{0}C_{n}+\sum_{i=1}^{n-1}C_{i}V}{Q}$$

*P_n_* represents the percentage of drug released at each time point;
*N* represents the concentration of released repellent in the system at each time point;
*V*_0_ is the total volume of the solution, fixed at 1000 mL;
*V* is the volume collected each time, fixed at 5 mL;
*Q* is the mass of the repellent adhered to the fabric (calculated based on the working solution ratio).

For each time point, after the percentage of repellent release is determined, the concentration of the released repellent in the system is calculated as follows, and the drug release profile is then plotted based on the data:

$$N=\frac{P_{n}Q}{V}$$

### 2.5. Nanoparticle Adhesion Testing

Cut the untreated fabric into 5 × 5 cm squares and weigh them, recording the initial weight as *W*_0_. After electrospray finishing, weigh the fabric again and record the weight as *W_a_*. Place the finished fabric in a beaker, add 1000 mL of tap water at 20 ± 5 °C, and set the stirring rate to 50 r/min to simulate household textile washing conditions. Every 30 min of stirring, remove the fabric sample and dry it for 20 min, ensuring complete dryness each time. This entire process constitutes one wash cycle. Repeat this process for 10 wash cycles in accordance with standard textile durability testing (e.g., AATCC Test Method 61) to simulate typical usage and laundering conditions. After 10 cycles and drying, weigh the fabric and record the weight as W_10_. The nanoparticle retention rate is calculated as follows:

$$\begin{array}{c} W_{∆}=W_{a}-W_{0} \end{array}$$

$$\begin{array}{c} W_{x}=W_{10}-W_{0} \end{array}$$

$$\begin{array}{c} R=\frac{W_{x}}{W_{∆}}\times 100\% \end{array}$$

*W*_Δ_ represents the theoretical initial adhesion weight of electrosprayed particles;
*W*_0_ represents the initial weight of the fabric;
*W_a_* represents the weight of the fabric after electrospraying;
*W*_10_ represents the weight of the electrospray fabric after 10 wash cycles;
*W_x_* represents the retained particle weight after 10 wash cycles;
*R* represents the particle retention rate after 10 wash cycles.

### 2.6. Biocompatibility and Safety Examination

Sensitization Test: Apply the test sample directly onto a 2.5 cm × 2.5 cm depilated animal skin area and keep it occluded for 6 ± 0.5 h. After removal, observe the application site for erythema and edema at 24 ± 2 h and 48 ± 2 h, and evaluate it according to the Magnusson and Kligman grading scale.

Irritation/Corrosion Test: Apply the test sample and control substance directly onto a 2.5 cm × 2.5 cm area of depilated animal skin and leave it for 4 h. Observe and record the skin condition at the application site at 1 ± 0.1 h, 24 ± 2 h, 48 ± 2 h, and 72 ± 2 h after removal. Document signs of skin irritation (reversible damage) and corrosion (irreversible damage), such as erythema, edema, ulceration, hemorrhage, and scabbing, and assign scores accordingly.

Cytotoxicity Test: The test samples were prepared at four different concentrations (100%, 50%, 25%, and 12.5%) to treat L929 cells and human dermal fibroblasts for 24 h. After that, 50 μL of MTT solution was added to each well and incubated at 37 °C for 2 h. The MTT solution was then removed, and 100 μL of isopropanol was added. Absorbance was measured at a wavelength of 570 nm to calculate cell viability.

### 2.7. Detection of Jellyfish Repellency Efficiency

To ensure statistical significance, 10 healthy jellyfish of a similar size were placed in the central area and allowed to swim freely for 5 min to allow them to stabilize in the new environment. Zone division was performed based on behavioral ecology studies to assess repellency and avoidance. The repellent-treated fabric was introduced into the designated repellent zone, and the partition was removed. The distribution of jellyfish across the three zones was recorded at 1 min intervals. Simultaneously, high-resolution video and photographic documentation were used to track the number of jellyfish in each zone and quantify those exhibiting loss of mobility. The repellency rate was calculated as follows:

$$P=\frac{\left(M+K\right)}{N}\times 100\%$$

*P*: Repellency rate (%);
*M*: Number of jellyfish that escaped to zones outside the repellent-treated area by the end of the test;
*K*: Number of jellyfish that became immobilized by the end of the test;
*N*: Total number of jellyfish across all three groups by the end of the test.

### 2.8. Statistical Analysis

Data are presented as the mean ± standard error (SE). Two-way ANOVA was used for comparisons between multiple independent samples, with *p* < 0.05 considered statistically significant.

## 3. Results

### 3.1. Implementation of Multi-Fluid Electrospraying

**Figure 1:**
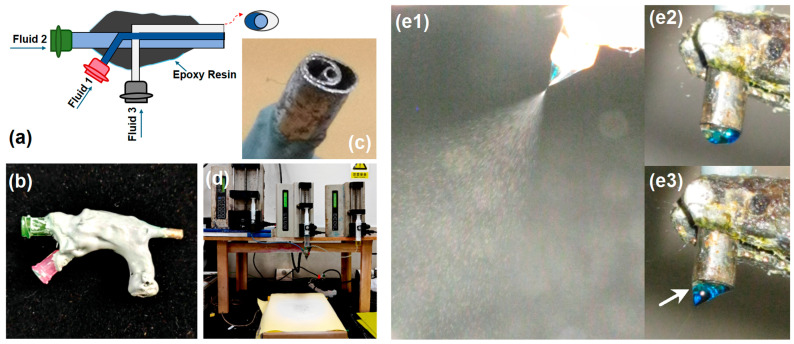
The process of the synthesis and spraying of copper ion-loaded multicompartmental nanoparticles. (**a**) Schematic diagram of the cross-section of the “Concentric Heterogeneous Sheath” nozzle; (**b**,**c**) physical photographs; (**d**) complete electrostatic spraying setup; (**e1**–**e3**) three typical stages of “Concentric Heterogeneous Sheath” electrostatic spray atomization. (**e1**) is a typical electrospraying process, which include the compound Taylor cone, the convergent point, and the atom-ization region. (**e2**) is a compound droplet before the application of high voltage. (**e3**) is a digital image of the compound Taylor cone for initiating the atomization process. Arrow indicates structure of nanoparticle.

As indicated in Figure 1a, a patented spinneret [28] was employed for the implementation of trifluid electrospraying to set the nanoparticles on the polyester fabrics. The spinneret can simultaneously guide three types of working fluids to its nozzle in an organized manner, and, using this template, multiple-chamber structural nanoparticles can be created in a single-step and a straightforward manner. An entire image and a digital photo of the nozzle are shown in Figure 1b,c. The apparatus, centered around the spinneret, has three pumps, a collector, and a power supply, as shown in Figure 1d. A typical electrospraying process and its Taylor cone are exhibited in Figure 1(e1–e3) and the top-right inset.

### 3.2. Characterization of Multicompartmental Nanoparticles

**Figure 2:**
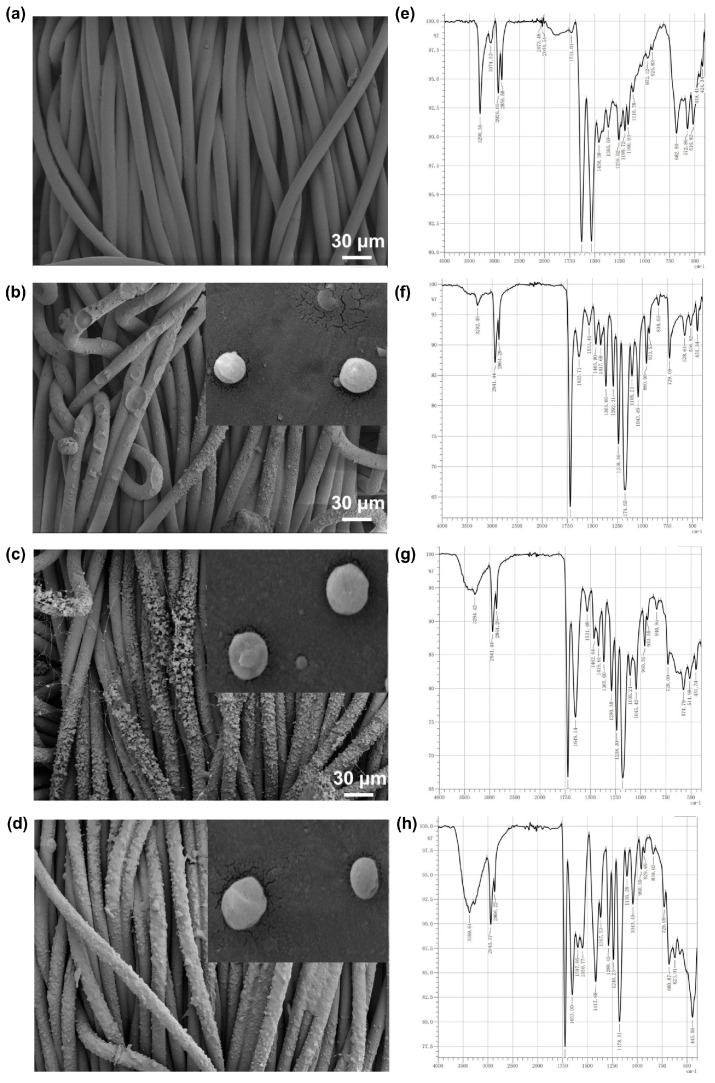
Characterization of Cu^2+^-loaded nanoparticles. SEM figures of (**a**) blank fabric, (**b**) copper chloride-loaded fabric, (**c**) copper sulfate-loaded fabric and (**d**) copper acetate-loaded fabric; FT-IR patterns of (**e**) blank fabric and the fabric coated with (**f**) copper chloride, (**g**) copper sulfate, and (**h**) copper acetate-loaded nanoparticles.

The successful synthesis and coating application of the nanoparticles, as confirmed via SEM analysis, indicate the formation of a uniform and well-defined multicompartmental structure that is crucial in controlled drug release. The size of copper chloride, copper sulfate, and copper acetate nanoparticles is 3.15 μm, 2.67 μm, and 2.28 μm, respectively (Figure 2a–d). The variation in nanoparticle sizes is attributed to differences in the solubility and crystallization behavior of the copper salts. Smaller particles may facilitate faster initial release, while larger ones contribute to sustained release, collectively optimizing repellency duration. The FT-IR patterns obtained from the samples showed that Cu^2+^ was successfully anchored through coordination with hydroxyl groups on the fabric fibers. Characteristic infrared absorption peaks were observed in comparison with the blank original fabric sample, providing direct confirmation of anchoring. For copper chloride, although its anion (Cl^−^) is infrared-inactive, the significant changes in the O–H infrared characteristic peaks resulting from the interaction between Cu^2+^ and the fibers also demonstrated successful anchoring. Moreover, all components were successfully anchored (Figure 2e–h).

### 3.3. Drug Release Profile of Cu^2+^-Loaded Multicompartmental Nanoparticles

**Figure 3:**
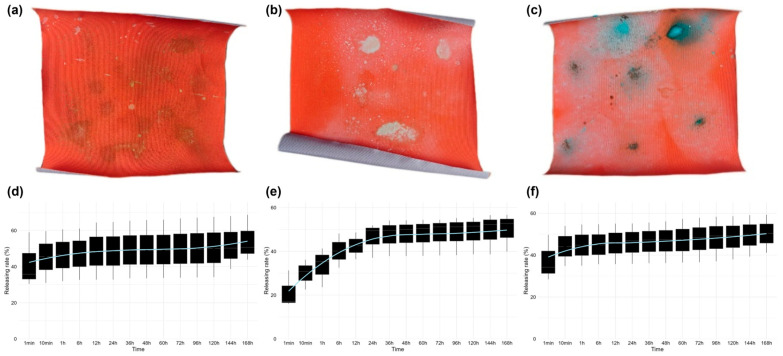
The drug release profile of multicompartmental nanoparticles. Images of fabrics sprayed with (**a**) copper chloride-, (**b**) copper sulfate-, and (**c**) copper acetate-loaded nanoparticles; the releasing curves of copper ions from (**d**) copper chloride-, (**e**) copper sulfate-, and (**f**) copper acetate-loaded nanoparticles over 168 h. Boxplots show the distribution of releasing rates at each time point, with the center line indicating the median, the box representing the interquartile range (IQR), and whiskers extending to 1.5 × IQR. The blue line represents the mean releasing rate at each time point.

Drug release testing demonstrated controlled and water-sensitive release of Cu^2+^ from the nanoparticles, which mimics the physiological conditions of human skin contact with jellyfish and is crucial in maintaining the repellency effect over an extended period. Upon contact with simulated seawater, the fabric begins to release the marine repellent (with copper ions as the detection standard) within one minute, and the sustained release duration exceeds 168 h (Figure 3).

### 3.4. Adhesion Property of Multicompartmental Nanoparticles

The results indicate that after 10 wash cycles, the nanoparticle retention rates for all three fabrics loaded with marine repellent nanoparticles remain above 70%. The theoretical total released amounts of copper chloride, copper sulfate, and copper acetate ions are 1.14 mg, 8.90 mg, and 9.57 mg, respectively (Table 1). The adhesion testing verified the robustness of the coating layer, ensuring its durability under various mechanical and environmental conditions.

### 3.5. Biocompatibility of the Fabric Coated with Cu^2+^-Loaded Multicompartmental Nanoparticles

In the skin sensitization test, no observable allergic reactions such as erythema or edema were detected in any experimental animals following the induction and challenge phases with the test sample. The observed responses were consistent with those in the negative control group (Table 2, Tables S1 and S2). In the skin irritation test, no erythema or edema was observed on the localized skin of the experimental animals during the observation period after the application of the test sample. The Primary Irritation Index (PII) calculated according to the Draize scoring standard was 0 (Table 3, Tables S3 and S4). Furthermore, the in vitro MTT assay results for the cytotoxicity of the extract toward L929 cells and human dermal fibroblasts showed that even at 100% concentration, the cell viability remained above the standard requirement threshold (above 70%), further confirming the absence of irritation (Table 4, Tables S5 and S6). In conclusion, under the conditions of this study, the test sample demonstrated no potential skin sensitization and no skin irritation.

### 3.6. Jellyfish Repellency Efficiency of the Fabric Coated with Cu^2+^-Loaded Multicompartmental Nanoparticles

**Figure 4:**
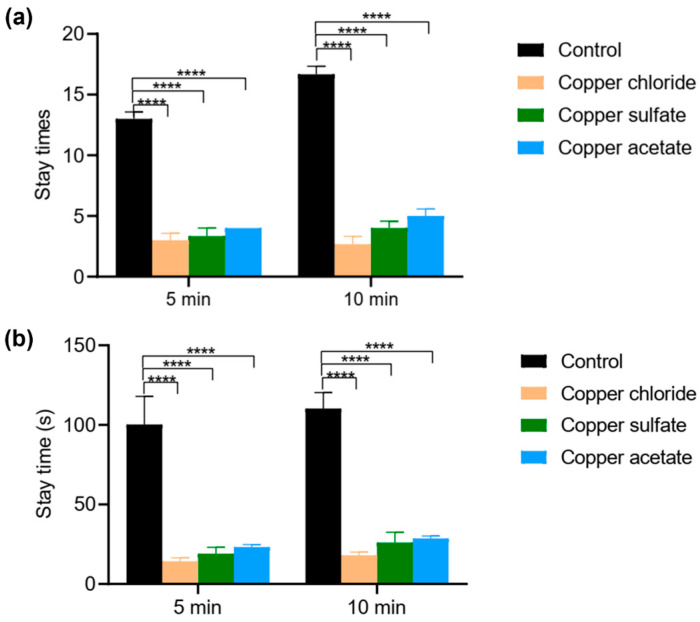
Repellency efficiency of different fabrics. (**a**) Stay times of jellyfish on the fabrics over 5 min and 10 min; (**b**) stay time of jellyfish on the fabrics over 5 min and 10 min. **** *p* < 0.0001.

Upon contact with the repellent fabric, jellyfish exhibited immediate avoidance behavior, including rapid tentacle retraction and directional movement away from the fabric. Some jellyfish lost vitality and became immobile after being affected by repellents upon contact with the fabric. Compared to the control group, sprayed fabric tests showed a reduction in the number and duration of stays. In particular, the significant decrease in dwell time demonstrates that the fabric loaded with repellent still exhibits a notable behavioral repellent effect on jellyfish (Figure 4). Our jellyfish repellency efficiency tests allowed a comprehensive evaluation of the nanoparticle-sprayed waterproof fabric, which validated the reduction in jellyfish stings, demonstrating the effectiveness of the coating in a natural environment, despite the difference compared to open-sea testing.

## 4. Discussion

Pharmaceutics is an interdisciplinary applied scientific field. It is continuously fed by advances in modern science and technology [29,30]. Electrohydrodynamic atomization methods, which exploit electrostatic energy to treat working fluids for material conversions, include electrospinning, electrospraying, and e-jetting writing and printing [31,32,33,34,35]. These new techniques have unique capabilities in terms of creating complex nanostructures in a straightforward manner [36,37]. Compared with the adjacent technique of electrospinning, electrospraying has received very limited attention. Thus, here, trifluid electrospraying was introduced as a nano-finishing method to develop novel formulations for “efficacious, safe and convenient” drug delivery, as well as new types of fabric for a specific application.

Jellyfish stings represent a significant and persistent risk to the safety of divers, fishers, and other underwater personnel, with potential consequences ranging from severe pain to life-threatening systemic reactions. While personal protective equipment remains the primary defense strategy, ongoing research has predominantly concentrated on enhancing the physical barrier properties of protective suits, such as their puncture resistance [4]. This approach, however, creates a notable market gap for solutions that provide active repellency. Addressing this limitation, our study introduces a paradigm shift by developing a novel jellyfish-repellent system based on Cu^2+^-loaded multicompartmental nanoparticles. This technology moves beyond mere physical blocking and offers an innovative mechanism for proactive jellyfish deterrence, establishing a new direction for the development of next-generation protective gear.

Our findings demonstrate the novel garment’s superiority over traditional protection methods [24]. The core innovation of this work lies in the unique multicompartmental architecture of the nanoparticles. Unlike conventional single-compartment nanocarriers, which often suffer from rapid, uncontrolled release of active agents, our designed system enables precise, sustained release of copper ions [38]. This controlled-release mechanism is critical in ensuring long-lasting protection, significantly extending the operational window for users engaged in prolonged marine activities and reducing the dependency on the frequent and often impractical reapplication of repellents. The release is triggered by the hydration and swelling of the polymer matrix in seawater; the ionic strength and water content facilitate the diffusion of Cu^2+^ ions, enabling a controlled and sustained release. The repellency effectiveness is primarily governed by the controlled release of Cu^2+^ rather than rapid changes in particle size, which remains relatively stable during release. Excellent adhesion to fabric substrates and the maintenance of the controlled release profile even after sustained friction and prolonged water immersion confirm that the coating can withstand the demanding conditions of harsh marine environments. Anchoring refers to the coordination bonding between Cu^2+^ and hydroxyl groups on the fabric, which enhances the stability of the ions on the fabric surface, prolonging their retention and release profile.

A further innovative aspect of this research is the strategic selection of the active repellent ingredient. Approaches to jellyfish repellent development have been largely conservative, with a strong focus on natural extracts [3,39]. While ecologically appealing, these compounds frequently lack the potency and durability required for reliable, long-term protection. In contrast, this study showcases an innovative application of copper ions, a well-documented biocide that acts as a highly effective repellent agent against jellyfish. The combination of the proven biocidal properties of copper with the advanced delivery capabilities of multicompartmental nanoparticles represents a significant conceptual and methodological leap, offering a more potent and durable alternative to the existing options.

To fully exploit the potential of this Cu^2+^-loaded nanoparticle coating, future research will take several strategic paths. The optimization of nanoparticle synthesis is paramount in achieving a cost-effective manufacturing process without compromising the functional integrity of the coating. Concurrently, expanding the scope of biological efficacy testing to assess repellency against a wider variety of jellyfish species will be essential in demonstrating broad-spectrum applicability. These concerted efforts will not only enhance the coating’s effectiveness and commercial sustainability but also broaden its potential applications across various marine industries. Moreover, this research will promote the integration of sophisticated nanotechnology into the field of personal protective equipment, paving the way for future exploration of smart, responsive material solutions for marine safety and beyond. While acute biocompatibility tests showed no adverse effects, future studies should include chronic exposure assessments to evaluate long-term safety under repeated-use conditions.

Nanostructures represent the frontiers of nanoscience and nanoengineering [40,41]. Among various types of complex nanostructures, core–shell and Janus structures are the most popular [42,43]. This is due to their effectiveness in developing novel functional nanomaterials based on concrete process–structure–property relationships [44,45]. The tri-chamber structure reported here is a combination of core–shell and Janus architectures. It is expected to offer the advantages of both core–shell and Janus nanostructures and thus will be more effective in developing multi-functional nanomaterials, including advanced nano drug delivery systems. Tri-chamber combined nanostructures can comprise electrosprayed nanoparticles, electrospun nanofibers [46,47], or beads-on-a-string nanoproducts.

## 5. Conclusions

This study successfully demonstrates a novel and effective strategy for jellyfish repellency through the synergy of copper ion-loaded multicompartmental nanoparticles, which are set on waterproof fabrics through a multi-fluid electrospraying process. The developed repellent coatings address critical limitations of traditional passive suits, offering active jellyfish repellency effects with good biocompatibility and safety. This work not only represents a significant advancement in the safety of underwater personnel but also establishes a new framework for applying advanced functional nanomaterials to protective textiles.

## Figures and Tables

**Table 1:** Adhesion property of Cu^2+^-loaded nanoparticles on fabrics.

Sample	*W*_0_ (g)	*W_a_* (g)	*W*_Δ_ (g)	*W*_10_ (g)	*W_x_* (g)	*R* (%)
Copper chloride (*n* = 3)	0.6389	0.7413	0.1024	0.7149	0.076	74.22
	0.5628	0.6286	0.0658	0.6122	0.0494	75.08
	0.6058	0.7192	0.1134	0.6889	0.0831	73.28
Copper sulfate (*n* = 3)	0.5883	0.665	0.0767	0.6374	0.0491	64.02
	0.6763	0.7827	0.1064	0.7655	0.0892	83.83
	0.6898	0.7381	0.0483	0.7235	0.0337	69.77
Copper acetate (*n* = 3)	0.6544	0.7848	0.1304	0.7722	0.1178	90.34
	0.6667	0.697	0.0303	0.6858	0.0191	63.04
	0.6493	0.7753	0.126	0.7565	0.1072	85.08

**Table 2:** Skin sensitization reaction of copper chloride-coated fabric.

Group	Animal ID (Weight/g)	Skin Elicitation Phase Grade		Positive Elicitation Rate (%)
		24 h	48 h	
Blank control	1 (351)	0	0	0
	2 (366)	0	0	
	3 (312)	0	0	
	4 (363)	0	0	
	5 (329)	0	0	
Treat	1 (318)	0	0	0
	2 (342)	0	0	
	3 (329)	0	0	
	4(327)	0	0	
	5 (340)	0	0	
	6 (347)	0	0	
	7 (325)	0	0	
	8 (316)	0	0	
	9 (311)	0	0	
	10 (340)	0	0	
Positive control	1 (349)	2	2	100
	2 (328)	2	2	
	3 (338)	2	2	
	4 (357)	1	1	
	5 (366)	1	1	
	6 (324)	2	1	
	7 (320)	1	1	
	8 (348)	2	2	
	9 (359)	1	1	
	10 (364)	2	2	

**Table 3:** Skin irritation reaction rating scale of copper chloride-coated fabric.

Animal ID (Weight/g)	1 h				24 h				48 h				72 h			
	Treatment		Control		Treatment		Control		Treatment		Control		Treatment		Control	
	Erythema/Edema	Total	Erythema/Edema	Total	Erythema/Edema	Total	Erythema/Edema	Total	Erythema/Edema	Total	Erythema/Edema	Total	Erythema/Edema	Total	Erythema/Edema	Total
1 (2410)	0/0	0	0/0	0	0/0	0	0/0	0	0/0	0	0/0	0	0/0	0	0/0	0
2 (2490)	0/0	0	0/0	0	0/0	0	0/0	0	0/0	0	0/0	0	0/0	0	0/0	0
3 (2630)	0/0	0	0/0	0	0/0	0	0/0	0	0/0	0	0/0	0	0/0	0	0/0	0
Score mean	0.00		0.00		0.00		0.00		0.00		0.00		0.00		0.00	

**Table 4:** Cytotoxicity of copper chloride-coated fabric.

Group	100% Test Solution of the Sample	50% Test Solution of the Sample	25% Test Solution of the Sample	12.5% Test Solution of the Sample	Negative Control	Positive Control	Blank Control
Mean	0.3955	0.4032	0.4190	0.4185	0.4262	0.4262	0.3730
SD	0.0387	0.0339	0.0258	0.0298	0.0340	0.0340	0.0254
Survival rate (%)	106.03	108.09	112.33	112.2	114.26	114.26	100.00

## Data Availability

All the research students’ data can be obtained from the corresponding author.

## Supplementary Materials

The following supporting information can be downloaded at: https://www.mdpi.com/article/10.3390/pharmaceutics18010047/s1, Table S1: Skin sensitization reaction of copper sulfate-coated fabric; Table S2: Skin sensitization reaction of copper acetate-coated fabric; Table S3: Skin irritation reaction rating scale of copper sulfate-coated fabric; Table S4: Skin irritation reaction rating scale of copper acetate-coated fabric; Table S5: Cytotoxicity of copper sulfate-coated fabric; Table S6: Cytotoxicity of copper acetate-coated fabric.

## Abbreviations

PCL	Polycaprolactone
TPU	Thermoplastic polyurethane
TFE	Trifluoroethanol
DMF	Dimethylformamide
DCM	Dichloromethane
THF	Tetrahydrofuran
SEM	Scanning electron microscopy
