# Supplementary material for: Waterproof Fabric with Copper Ion-Loaded Multicompartmental Nanoparticle Coatings for Jellyfish Repellency
Source: Pharmaceutics. 2025 Dec 30;18(1):47. doi: 10.3390/pharmaceutics18010047 (PMC12844905; doi:10.3390/pharmaceutics18010047)
Supplement: Supplementary file 1 [file pharmaceutics-18-00047-s001.zip › Supplementary materials.docx]

**Supplementary Materials**

**Waterproof Fabric with Copper Irons-Loaded Multicompartmental Nanoparticle Coatings for Jellyfish Repellency**

Bo Wang,^1†^ Ruiqian Yao, ^1†^ Muzi Yang,^2†^ Liang Xiao, ^2^ Haixia Zhao, ^1^ Dengguang Yu,^3^ Lin Du, ^1*^ Shuaijun Zou, ^1*^ Yuanjie Zhu ^1*^

^1^Department of Dermatology, Naval Medical Center, Naval Medical University, Shanghai 200052, China.

^2^ Faculty of Naval Medicine, Naval Medical University, Shanghai 200433, China.

^3^ School of Materials & Chemistry, University of Shanghai for Science and Technology, Shanghai 200093, China

^†^These authors contributed equally to this work.

Corresponding author:

Lin Du, Shuaijun Zou & Yuanjie Zhu, Department of Dermatology, Naval Medical Center, Naval Medical University, Shanghai 200052, China. Email: [lynnie_du@126.com.(L.D.)](mailto:lynnie_du@126.com.(L.D.)); [sjzou@smmu.edu.cn.(S.Z.)](mailto:sjzou@smmu.edu.cn.(S.Z.)); [zhuyj@smmu.edu.cn.(Y.Z.)](mailto:zhuyj@smmu.edu.cn.(Y.Z.))

**Table S1 Skin sensitization reaction of** **copper sulfate-coated fabric**

| Group | Animal ID (weight/g) | Skin elicitation phase grade | | Positive Elicitation Rate (%) |
| --- | --- | --- | --- | --- |
|  |  | 24h | 48h |  |
| Blank control | 1 (328) | 0 | 0 | 0 |
|  | 2 (340) | 0 | 0 |  |
|  | 3 (317) | 0 | 0 |  |
|  | 4 (366) | 0 | 0 |  |
|  | 5 (328) | 0 | 0 |  |
| Treat | 1 (328) | 0 | 0 | 0 |
|  | 2 (360) | 0 | 0 |  |
|  | 3 (322) | 0 | 0 |  |
|  | 4(357) | 0 | 0 |  |
|  | 5 (311) | 0 | 0 |  |
|  | 6 (349) | 0 | 0 |  |
|  | 7 (335) | 0 | 0 |  |
|  | 8 (329) | 0 | 0 |  |
|  | 9 (318) | 0 | 0 |  |
|  | 10 (324) | 0 | 0 |  |
| Positive control | 1 (349) | 2 | 2 | 100 |
|  | 2 (328) | 2 | 2 |  |
|  | 3 (338) | 2 | 2 |  |
|  | 4 (357) | 1 | 1 |  |
|  | 5 (366) | 1 | 1 |  |
|  | 6 (324) | 2 | 1 |  |
|  | 7 (320) | 1 | 1 |  |
|  | 8 (348) | 2 | 2 |  |
|  | 9 (359) | 1 | 1 |  |
|  | 10 (364) | 2 | 2 |  |

**Table S2 Skin sensitization reaction of copper acetate-coated fabric**

| Group | Animal ID (weight/g) | Skin elicitation phase grade | | Positive Elicitation Rate (%) |
| --- | --- | --- | --- | --- |
|  |  | 24h | 48h |  |
| Blank control | 1 (351) | 0 | 0 | 0 |
|  | 2 (340) | 0 | 0 |  |
|  | 3 (318) | 0 | 0 |  |
|  | 4 (312) | 0 | 0 |  |
|  | 5 (340) | 0 | 0 |  |
| Treat | 1 (328) | 0 | 0 | 0 |
|  | 2 (360) | 0 | 0 |  |
|  | 3 (322) | 0 | 0 |  |
|  | 4(357) | 0 | 0 |  |
|  | 5 (311) | 0 | 0 |  |
|  | 6 (349) | 0 | 0 |  |
|  | 7 (335) | 0 | 0 |  |
|  | 8 (329) | 0 | 0 |  |
|  | 9 (318) | 0 | 0 |  |
|  | 10 (324) | 0 | 0 |  |
| Positive control | 1 (349) | 2 | 2 | 100 |
|  | 2 (328) | 2 | 2 |  |
|  | 3 (338) | 2 | 2 |  |
|  | 4 (357) | 1 | 1 |  |
|  | 5 (366) | 1 | 1 |  |
|  | 6 (324) | 2 | 1 |  |
|  | 7 (320) | 1 | 1 |  |
|  | 8 (348) | 2 | 2 |  |
|  | 9 (359) | 1 | 1 |  |
|  | 10 (364) | 2 | 2 |  |

**Table S3 Skin irritation reaction rating scale of copper sulfate-coated fabric**

| Animal ID (weight/g) | 1h | | | | 24h | | | | 48h | | | | 72h | | | | |
| --- | --- | --- | --- | --- | --- | --- | --- | --- | --- | --- | --- | --- | --- | --- | --- | --- | --- |
|  | Treat | | Control | | Treat | | Control | | Treat | | Control | | Treat | | Control | |  |
|  | Erythema/Edema | Total | Erythema/Edema | Total | Erythema/Edema | Total | Erythema/Edema | Total | Erythema/Edema | Total | Erythema/Edema | Total | Erythema/Edema | Total | Erythema/Edema | Total |  |
| 1 (2560) | 0/0 | 0 | 0/0 | 0 | 0/0 | 0 | 0/0 | 0 | 0/0 | 0 | 0/0 | 0 | 0/0 | 0 | 0/0 | 0 |  |
| 2 (2590) | 0/0 | 0 | 0/0 | 0 | 0/0 | 0 | 0/0 | 0 | 0/0 | 0 | 0/0 | 0 | 0/0 | 0 | 0/0 | 0 |  |
| 3 (2410) | 0/0 | 0 | 0/0 | 0 | 0/0 | 0 | 0/0 | 0 | 0/0 | 0 | 0/0 | 0 | 0/0 | 0 | 0/0 | 0 |  |
| Score mean | 0.00 | | 0.00 | | 0.00 | | 0.00 | | 0.00 | | 0.00 | | 0.00 | | 0.00 | |  |

**Table S4 Skin irritation reaction rating scale of copper acetate-coated fabric**

| Animal ID (weight/g) | 1h | | | | 24h | | | | 48h | | | | 72h | | | | |
| --- | --- | --- | --- | --- | --- | --- | --- | --- | --- | --- | --- | --- | --- | --- | --- | --- | --- |
|  | Treat | | Control | | Treat | | Control | | Treat | | Control | | Treat | | Control | |  |
|  | Erythema/Edema | Total | Erythema/Edema | Total | Erythema/Edema | Total | Erythema/Edema | Total | Erythema/Edema | Total | Erythema/Edema | Total | Erythema/Edema | Total | Erythema/Edema | Total |  |
| 1 (2660) | 0/0 | 0 | 0/0 | 0 | 0/0 | 0 | 0/0 | 0 | 0/0 | 0 | 0/0 | 0 | 0/0 | 0 | 0/0 | 0 |  |
| 2 (2420) | 0/0 | 0 | 0/0 | 0 | 0/0 | 0 | 0/0 | 0 | 0/0 | 0 | 0/0 | 0 | 0/0 | 0 | 0/0 | 0 |  |
| 3 (2530) | 0/0 | 0 | 0/0 | 0 | 0/0 | 0 | 0/0 | 0 | 0/0 | 0 | 0/0 | 0 | 0/0 | 0 | 0/0 | 0 |  |
| Score mean | 0.00 | | 0.00 | | 0.00 | | 0.00 | | 0.00 | | 0.00 | | 0.00 | | 0.00 | |  |

**Table S5 Cytotoxicity of copper sulfate-coated fabric**

| Group | 100% Test solution of the sample | 50% Test solution of the sample | 25% Test solution of the sample | 12.5% Test solution of the sample | Negative control | Positive control | Blank control |
| --- | --- | --- | --- | --- | --- | --- | --- |
| Mean | 0.5564 | 0.5687 | 0.565 | 0.575 | 0.6137 | 0.0765 | 0.5119 |
| SD | 0.0319 | 0.0481 | 0.0421 | 0.0364 | 0.0576 | 0.0135 | 0.0149 |
| Survival rate (%) | 108.69 | 111.09 | 110.37 | 112.32 | 119.88 | 14.94 | 100.00 |

**Table S6 Cytotoxicity of copper acetate-coated fabric**

| Group | 100% Test solution of the sample | 50% Test solution of the sample | 25% Test solution of the sample | 12.5% Test solution of the sample | Negative control | Positive control | Blank control |
| --- | --- | --- | --- | --- | --- | --- | --- |
| Mean | 0.4930 | 0.5025 | 0.4953 | 0.5170 | 0.5105 | 0.0900 | 0.4637 |
| SD | 0.0392 | 0.0471 | 0.0376 | 0.0146 | 0.0222 | 0.0175 | 0.0244 |
| Survival rate (%) | 106.31 | 108.36 | 106.82 | 111.49 | 110.09 | 19.41 | 100.00 |
